# Biodegradable Polymeric Architectures via Reversible Deactivation Radical Polymerizations

**DOI:** 10.3390/polym10070758

**Published:** 2018-07-09

**Authors:** Fengyu Quan, Aitang Zhang, Fangfang Cheng, Liang Cui, Jingquan Liu, Yanzhi Xia

**Affiliations:** 1 College of Materials Science and Engineering, Institute for Graphene Applied Technology Innovation, Collaborative Innovation Centre for Marine Biomass Fibers, Materials and Textiles of Shandong Province, Qingdao University, Qingdao 266071, China; quanfengyu@qdu.edu.cn (F.Q.); qingdauniversity@163.com (A.Z.); 13646483925@163.com (F.C.); 2 College of Materials Science and Engineering, Linyi University, Linyi 276000, China

**Keywords:** biodegradable, polymeric structures, reversible deactivation radical polymerizations

## Abstract

Reversible deactivation radical polymerizations (RDRPs) have proven to be the convenient tools for the preparation of polymeric architectures and nanostructured materials. When biodegradability is conferred to these materials, many biomedical applications can be envisioned. In this review, we discuss the synthesis and applications of biodegradable polymeric architectures using different RDRPs. These biodegradable polymeric structures can be designed as well-defined star-shaped, cross-linked or hyperbranched via smartly designing the chain transfer agents and/or post-polymerization modifications. These polymers can also be exploited to fabricate micelles, vesicles and capsules via either self-assembly or cross-linking methodologies. Nanogels and hydrogels can also be prepared via RDRPs and their applications in biomedical science are also discussed. In addition to the synthetic polymers, varied natural precursors such as cellulose and biomolecules can also be employed to prepare biodegradable polymeric architectures.

## 1. Introduction

Biodegradable polymers refer to a category of polymers that can be cleaved into small polymer fragments in vivo. The biodegradability endows these polymers with many special applications particularly in drug delivery, tissue regeneration and biotherapeutics [1,2,3]. Methods for the preparation of biodegradable polymers can be versatile. Voit and Lederer reviewed the synthesis and major characterizations of hyperbranched and highly branched polymer architectures using polycondensation, addition step-growth reaction and cycloaddition reactions, self-condensing vinyl polymerization and ring-opening multi-branching techniques [4]. The exploitation of “green” atom transfer radical polymerization (ATRP) and ring-opening polymerization (ROP) to design well-defined and eco-friendly polymeric materials such as biodegradable polymers, polymer brushes, nonionic polymeric surfactants, etc. was reviewed by Tsarevsky and Matyjaszewski [5]. Utilizing various polymers for fabricating the more complicated polymeric particles, e.g., micelles, vesicles and capsules, has also been well-documented [6,7,8]. Reversible deactivation radical polymerizations (RDRPs) is a relatively new polymerization technique but has already been well-explored. Due to its advantages over other techniques on the preparation of well-defined polymers with low molecular weight distributions, particularly in the preparation of versatile hyperbranched and multi-functional polymeric architectures, in this review, we mainly focus on discussing the preparation of versatile polymeric architectures via RDRPs.

### 1.1. Varied Polymeric Architectures

**Scheme 1:**
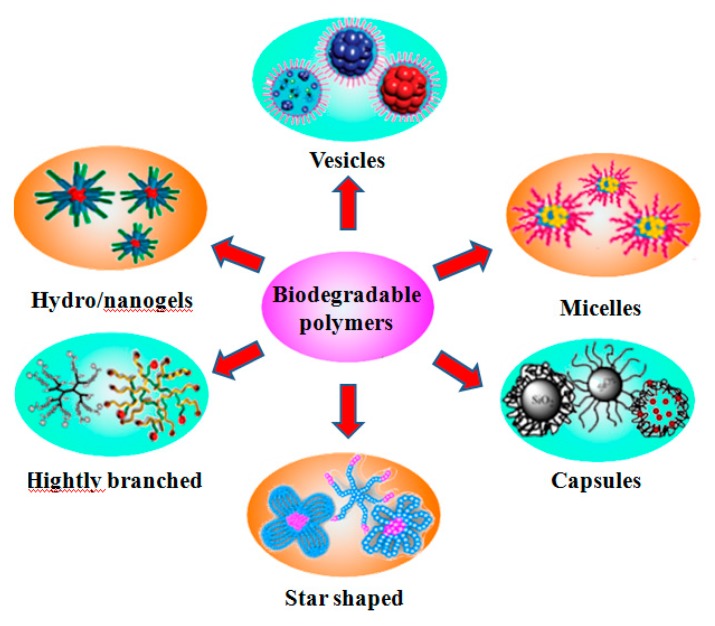
Schematic illustration for biodegradable polymeric architectures via reversible deactivation radical polymerizations.

Polymeric architectures are very versatile. Based on the composition, they can be homopolymers, or block, statistical, gradient and graft copolymers. Based on the structure, they can be designed as linear, multi-armed, comb-like, networks, and hyperbranched polymers. They can also be tailored with single, multi-, homo-, hetero- or multi-functionalities. These broad polymeric architectures can be fabricated into various complicated particles via either self-assembly or designed interactions, such as micelles, vesicles, capsules, hydrogels and nanogels (Scheme 1). Because RDRPs have controlled and living polymerization properties and the chain transfer agent (CTA) employed for the RDRPs can be flexibly designed, for instance, as linear, multi-armed or functional, they are convenient tools for the synthesis of the more complicated architectures. The combination of different RDRPs methods is usually the solution for generation of the more complicated polymeric architectures [9,10].

### 1.2. Reversible Deactivation Radical Polymerizations

**Figure 1:**
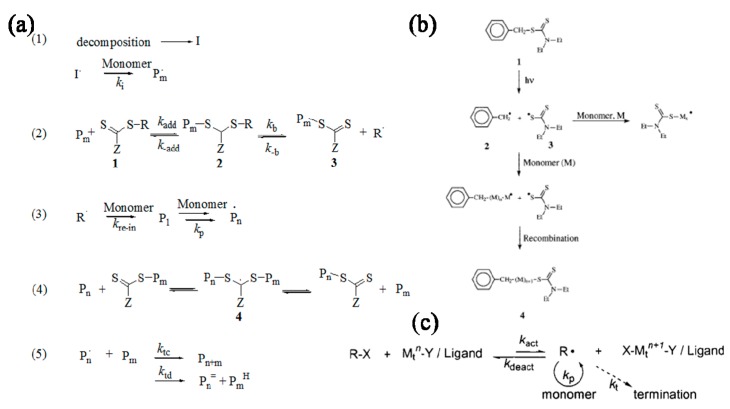
(**a**) Generally accepted mechanism for a RAFT polymerization. Copyright 2009, American Chemical Society; (**b**) The mechanism for NMRP polymerization. Copyright 2001, American Chemical Society; (**c**) Transition-metal-catalyzed ATRP. Copyright 2001, American Chemical Society.

In addition to ionic and coordination ring-opening polymerization [11,12], free radical polymerization RDRPs have been exploited extensively to generate multi-armed structures with predetermined molecular weights and narrow molecular weight distributions. ATRP [13,14], nitroxide mediated radical polymerization (NMRP) [15] and reversible addition fragmentation chain transfer (RAFT) polymerization [16,17,18,19,20] are the most explored RDRPs (Figure 1). ATRP is one of the most studied RDRPs and many articles have been published about this topic since its development in 1995 by Matyjaszewski [21,22]. ATRP is usually initiated by a halogenated organic species in the presence of a metal halide. The metal has a number of different oxidation states that allows it to attract a halide from the organohalide, creating a radical that then starts free radical polymerization. ATRP is an excellent tool for the synthesis of well-defined polymers, however the low solubility of metal halides may limit the catalyst availability and the residual catalyst among the as-prepared polymers may limit the applications in biological field and electronic devices [23]. RAFT polymerization was discovered by Rizzardo et al. only two decade ago, but has also been well-explored and -employed to synthesize polymers with predetermined molecular weight and narrow molecular weight distributions over a wide range of monomers. RAFT technique is suitable for polymerizing versatile monomers in different media, where solution (either in organic or aqueous media), emulsion and suspension polymerizations can be carried out for purposely generating functionalized polymers. These functional groups can also be exploited for further polymerization or further reaction to form complicated architectures. RAFT polymerization, in comparison with ATRP, can be undertaken without the introduction of metal ion catalysts, therefore, it will be a secure tool particularly in biological and electrical applications [17,24,25].

### 1.3. Necessity for Making Biodegradable Polymeric Architectures

Biodegradable polymeric architectures have many advantages that could be envisioned [26]. First, previous research revealed that polymers with high molecular weight over 50,000 g·mol^−1^ will exhibit significantly increased circulation time in the body since the glomerular filtration in the kidney has a molecular weight cut-off of about 50,000 g·mol^−1^ [27]. Biodegradable polymeric architectures tend to be cleaved into smaller fragments in vivo and subsequently excreted out of the body, which will greatly help clean the polymer fragments within the body. Second, biodegradable polymeric architectures will offer important applications in bio-therapeutics. For example, protein and peptide drugs hold great promise as therapeutic agents. However, most of these drugs can be degraded by proteolytic enzymes and rapidly cleared by the kidneys, resulting in a short circulating half-life. Fortunately, when polyethylene glycol chains are attached to protein and peptide drugs, their circulation time and pharmacokinetics can be significantly improved [28]. Third, another advantage is that when the biodegradable polymers are employed to fabricate nanoparticles as drug carriers, the drug release can be realized via the disintegration of polymeric nanoparticles upon biodegradation in vivo.

### 1.4. How to Confer Biodegradability to Polymeric Architectures

To confer biodegradability to polymers, they have to be designed with intra-linkers that can be cleaved by either physiological substances (e.g., glutathione) or enzymatic catalysis [29,30]. The biodegradable linkages can be tailored on the polymer backbones, on the side chains, on the cross-linking agents, etc. Several covalent linkages are biodegradable, e.g., the acetal linkage is acid labile [31]; the ester linkage is degradable upon hydrolysis [32,33]; disulfide bond is cleavable in the presence of glutathione (GSH), the most abundant intracellular thiol (0.2–10 mM) in most mammalian and many prokaryotic cells [34,35,36]; and polymers such as polycaprolactone (PCL) [37] and poly(amino acid)s [38] with polypeptide backbone can be degraded in biological environments by enzymes such as proteinases and peptidases.

### 1.5. Scope of the Review

This review discusses the synthesis of versatile biodegradable polymeric architectures that undergo biodegradation using the technique of RDRPs, and their biomedical applications, such as gene/drug delivery, controlled release, targeting biotherapeutics, nanomedicine and so on are also highlighted.

## 2. Biodegradable Polymeric Architectures

### 2.1. Well-Defined Star-Shaped Structures

Well-defined polymeric structures, e.g., star polymers, are of particular significance in biological applications such as drug delivery and bio-therapeutics [39]. Generally, star polymeric structures can be synthesized via “arm-first” or “core first” methodologies. The “arm-first” methodology can be used to generate multi-armed structures by either cross-linking the linear polymeric chains or post-polymerization conjugation of linear functionalized polymeric chains to a multi-functional core via chemo-selectively covalent coupling or non-covalent interactions, e.g., metal ion mediated coordination [9,40,41,42,43,44,45,46,47,48]. The “core first” strategy is more straightforward, and therefore has attracted an increasing interest for generating multi-armed polymeric architectures in a more controllable mode using multi-functional chain transfer agent [49,50,51,52,53,54]. Star polymers consisting of miktoarms have also been tailored to achieve different properties [10,42,55,56].

**Figure 2:**
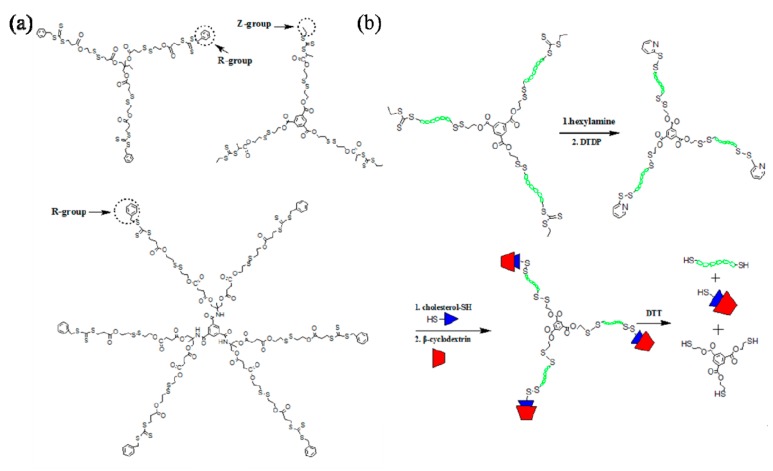
(**a**) Multi-armed RAFT agents with biodegradable disulfide intra-linkers: three-armed RAFT agent with *R*-group at the end of each arm; three-armed RAFT agent with *Z*-group at the end of each arm and six-armed RAFT agent with *R*-group at the end of each arm; (**b**) Post polymerization modification of the three-armed star polymers with RAFT active center at the end of each arm. Copyright 2009, American Chemical Society.

Multi-armed star polymeric architectures have attracted increasing interest due to their potential applications in a number of areas, e.g., encapsulation, sensing, catalysis, electronics, optics, biological engineering, coatings, additives, and drug and gene delivery [57,58]. In recent studies, Davis and coworkers successfully demonstrated the synthesis of three-armed star polymeric architectures using both “core first” and “arm first” methodologies to generate three-armed architecture containing biodegradable disulfide linkages. When “arm first” method was adopted, the linear polymer chain was tailored with thiol-reactive pyridyl disulfide groups, through which the linear chains were attached onto a tri-thiol functional core to afford three-armed star polymeric structure. At the same time, the “core first” technique was also utilized to generate the same three-armed star polymers from RAFT controlled polymerization using a trifunctional RAFT agent (Figure 2a). Gel permeation chromatography (GPC) and electrospray ionization (ESI) mass spectroscopy analysis evidenced the well-controlled RAFT polymerization which yielded well-defined three-armed star structures with polydispersity index (PDI) less than 1.28. The R group was designed at the end of the RAFT agent, through which the as-prepared polymer chains would sit outside of the RAFT active centers, that is, at the end of each arm. Further modification of the RAFT cores would risk polymeric chain loss. This design would compromise the application when modification of the trithiocarbonate or dithioester RAFT cores is required. To overcome this drawback, Davis and coworkers designed a three-armed RAFT agent via a condensation reaction between the *R*-group of the RAFT agent and a trifunctional core to afford a trifunctional RAFT agent with *Z*-groups at the end of each arm. The subsequent polymerizations of styrene and PEG-A using this RAFT agent generated three-armed polymeric structures with trithiocarbonate cores at the end of each arm, endowing the potential for further modifications through the RAFT cores (Figure 2b) [59]. Aminolysis of the trithiocarbonate cores and further reaction with dithiodipyridine (DTDP) yielded sulfhydryl groups and subsequently pyridyldisulfide (PDS) terminal groups, available for further reactions with any free thiol-tethered precursors. When the ends of the star polymers were modified with cholesterol groups, α-cyclodextrin (α-CD) groups were attached successfully via inclusion complexation. The generated architecture can be easily degraded in the presence of DTT due to the introduction of disulfide linkages. The methodology presented here can be a prototype research for post-polymerization modifications of various polymeric architectures prepared by RAFT mechanism. 

As an extension, a six-armed star architecture with disulfide intra-linkages on each arm was also synthesized using “core-first” methodologies, where a six-armed RAFT agent was synthesized first by attaching the RAFT agent via its *Z*-group to a core that has six RAFT active sites, followed by the RAFT mediated polymerization [49,60]. The PDIs of the six-armed star polymers with amphiphilic copolymer arms of poly(St-*b*-PEG-A) were less than 1.31 for the copolymers up to 80% conversion, indicating a well-controlled mechanism by RAFT. After cleavage in the presence of dl-Dithiothereitol (DTT), the PDI of the single-armed chains was measured to be 1.20 by GPC, in accordance with the successful living polymerization. It should be emphasized that a lower PDI is not necessarily indicative of instantaneous arm growth from all thiocarbonate sites [61], as the fragmentation of the initial RAFT functionality may not favor the initiating group (R-group). This may be a noticeable problem at very low conversions, but as conversion proceeds, and the main RAFT equilibrium is attained, this is unlikely to become a significant influence on the kinetics and/or architectures.

**Figure 3:**
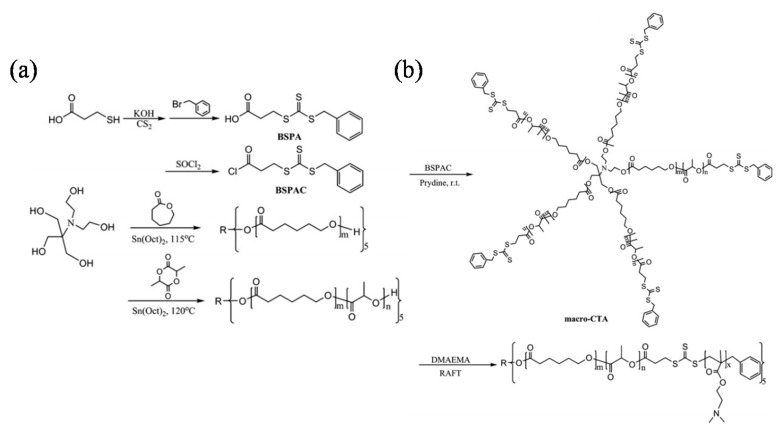
Synthesis of the star-block amphiphilic copolymer via ring-opening polymerization (**a**) and RAFT polymerization (**b**). Copyright 2010, Elsevier.

In Li’s study, biodegradable star-shaped poly(*ɛ*-caprolactone) and poly(*ɛ*-caprolactone-*b*-l-lactide) (5sPCL-*b*-PLLA) with five arms were synthesized by ring-opening polymerization (ROP) from an asymmetric core. Subsequently, a series of amphiphilic and double responsive star-block copolymers were synthesized by RAFT star polymerization of *N*,*N*-dimethylamino-2-ethyl methacrylate (DMAEMA) from the star-shaped macro-RAFT agent, which was prepared by attaching 3-benzylsulfanylthiocarbonylthiocarbonylsufanylpropionic acid (BSPA) to 5sPCL-*b*-PLLA using a simple two-step reaction sequence. GPC and ^1^H-NMR measurements demonstrated the polymerization courses are under control. The molecular weight of 5sPCL-*b*-PLLA-*b*-DMAEMA increased with increasing monomer conversion and the molecular weight distribution ranged 1.19–1.37. Spherical micelles with degradable core and pH and thermo-double sensitive shell were prepared from the aqueous medium of the amphiphilic star-shaped copolymers through a dialysis method. Both pH and thermal-responsive behaviors of the copolymer micelles in this study were investigated (Figure 3) [62]. In addition to the well-defined symmetrical multi-armed polymeric structures, biodegradable, penta-armed star-block copolymers were also synthesized via an asymmetric core by combination of ROP and RAFT polymerizations, where the five-armed macro-RAFT agent was prepared by ROP on each arm. 

To generate more complicated polymeric architectures, combined methods should be more effective [63]. Qiao and Wiltshire [64] synthesized the degradable polyester-based star polymers with a high level of functionality in the arms via the “arms first” approach using an acetylene-functional block copolymer macroinitiator. This was achieved by using 2-hydroxyethyl 2′-methyl-2′-bromopropionate to initiate the ROP of caprolactone monomer, followed by ATRP of a protected acetylene monomer, (trimethylsilyl) propargyl methacrylate. The hydroxyl end-group of the resulting block copolymer macroinitiator was subsequently cross-linked under ROP conditions using a bislactone monomer, 4,4′-bioxepanyl-7,7′-dione, to generate a degradable core cross-linked star (CCS) polymer with protected acetylene groups in the corona. After removal of trimethylsilyl-protecting groups the resulting pendent acetylene groups were then reacted with azide-functionalized linear polystyrene via a copper-catalyzed cycloaddition reaction between azide and acetylene functionalities. The “brush-like” arms could be cleaved via the hydrolysis of polyester star structure to generate molecular brushes. Combining RAFT polymerization with ATRP and hetero-Diels-Alder chemistry, Sinnwell et al. successfully prepared 12-armed star block copolymers. The biodegradable ester linkages between the arm and core confer the biodegradability to the generated polymeric architectures [65]. Star-shaped block copolymers with a biodegradable poly(lactide) core were also synthesized using RAFT polymerization combining copper-catalyzed Huisgen 1, 3-dipolar cycloaddition and thiol-ene Michael additions [66].

**Figure 4:**
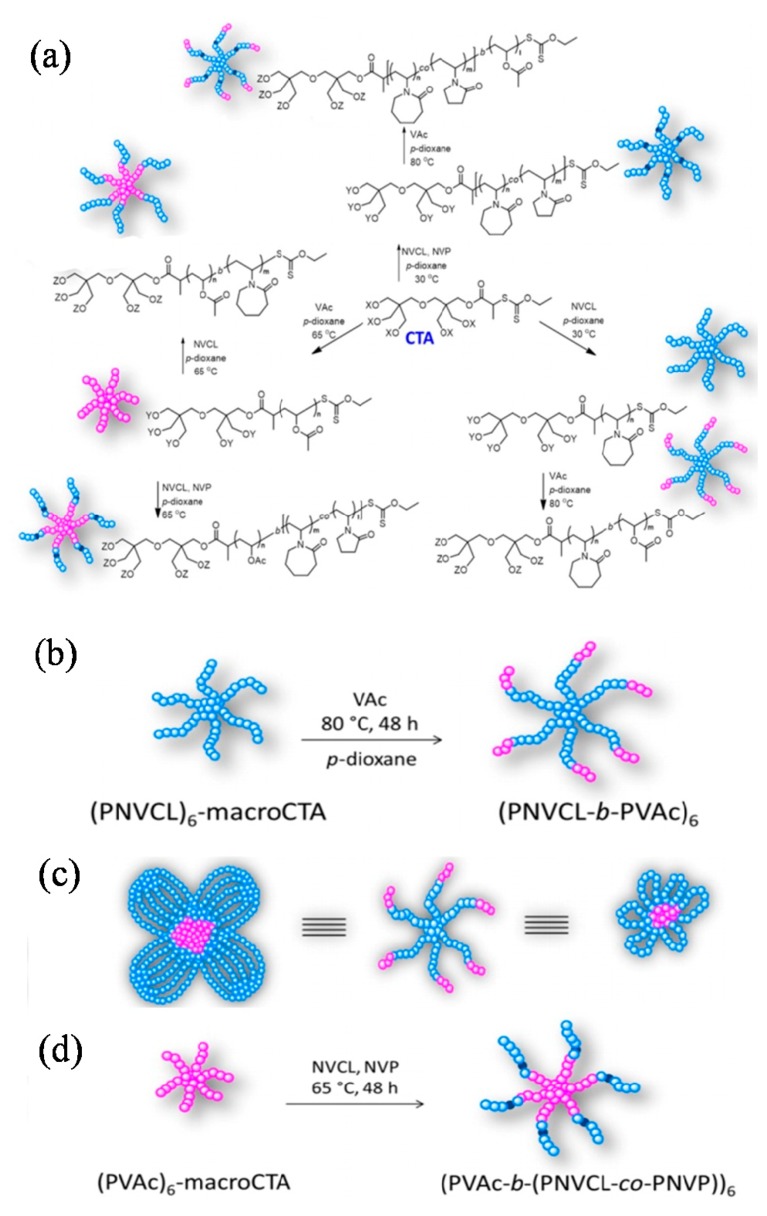
(**a**) Synthesis of hexafunctional star polymers and block copolymers based on PNVCL using amultifunctional xanthate as a RAFT agent; (**b**) Synthesis of star (PNVCL-*b*-PVAc)_6_ copolymers; (**c**) Self-assembly in aqueous solution of star (PNVCL-*b*-PVAc)_6_ block copolymers; (**d**) Synthesis of the star [PVAc-*b*-(PNVCL-*co*-PNVP)]_6_ block copolymers. The blue and red colors represent PNVCL and PVAc, respectively. Copyright 2017, Elsevier.

Few star-shaped thermoresponsive polymers with six arms were prepared via RAFT polymerization by Cortez-Lemus’s group. Star polymers with homopolymeric arms of poly(*N*-vinylcaprolactam) (PNVCL), copolymeric arms of poly(*N*-vinylcaprolactam-*co*-*N*-vinylpyrrolidone) (PNVCL-*co*-PNVP) and arms of block copolymers of poly(*N*-vinylcaprolactam-*b*-Vinyl acetate) (PNVCL-*b*-PVAc) and (PNVCL-*co*-PNVP)-*b*-PVAc were achieved by exploiting the R-RAFT synthetic methodology (or R-group approach), where the thiocarbonyl group is transferred to the polymeric chain end. Removing the xanthate group of the star polymers allowed for the introduction of specific functional groups at the ends of the star arms and resulted in an increase of the lower critical solution temperature (LCST) values. These star block copolymers could self-assemble into single flowerlike micelles, showing great stability in aqueous solution. Micellar aggregates of selected star polymers were used to encapsulate methotrexate showing their potential in the temperature controlled release of this antineoplasic drug (Figure 4) [67].

In another study reported by Qiao and Wiltshire, the synthesis of selectively degradable core cross-linked star polymers using ATRP and ROP was presented [68]. In their study, both the arms and the core can be designed to be biodegradable and selectively degraded. The arms were also designed to be the same or different. The multifunctional initiator, 2-hydroxyethyl 2′-methyl-2′-bromopropionate was used to synthesize degradable poly(*ε*-caprolactone) (PCL) and nondegradable polystyrene (PSt) and poly(methyl methacrylate) (PMMA) macro-initiators, which were subsequently cross-linked to generate core cross-linked star (CCS) polymers. By using the non-degradable divinylbenzene (DVB) and ethylene glycol dimethacrylate (EGDMA) as well as the degradable (4,4′-bioxepanyl-7,7′-dione (BOD) and 2,2-bis(*ε*-caprolactone-4-yl)propane (BCP) monomers to cross-link the different macro-initiators, a range of CCS polymers were synthesized where either the arm or the core domain can be selectively degraded. Hydrolysis of PCL/PMMA/EGDMA miktoarm CCS polymer resulted in CCS polymer with a reduced number of arms, whereas PSt/BOD core-degradable CCS polymer yielded the original linear PSt arms upon hydrolysis.

Similarly, Schramm et al. [69] also reported the synthesis of well-defined 4-, 6-, 8- and 12-armed star polymers with biodegradable PCL biodegradable cores, poly(*ε*-caprolactone)-*b*-poly(ethylene glycol) methacrylates (PEGMAs) using ATRP and ROP. These multi-armed star architectures exhibited unimolecular behavior and the capability of encapsulation of hydrophobic molecules, therefore they are potential candidates as hydrophobic anticancer drug carriers. Likewise, thermosensitive four armed triblock copolymers comprised of poly(*ε*-caprolactone), poly(olego(ethylene oxide) methacrylate) and poly(di(ethylene oxide)methyl ether methacrylate) segments were also synthesized by ATRP and ROP joint methods using a four armed initiator. These four armed polymeric structures were found to be able to self-assemble into spherical micelles which undergo reversible sol-gel transitions between room temperature (22 °C) and human body temperature (37 °C) [70]. Well-defined dendrimer-like star block copolymers up to 24 arms have also been successfully achieved by combination of ROP and ATRP using “core-first” methodology [71].

### 2.2. Cross-Linked (Highly Branched) Structures

RAFT polymerization can be a convenient tool for generating functionalized and biodegradable macro-monomers via wisely tailored RAFT agent. Davis and coworkers synthesized a novel AB_2_ macro-monomers bearing α-dithiobenzoate and ω-double pyridyl disulfide end-groups through a straightforward synthetic approach [72]. These monomers were prepared by RAFT polymerization, after which the α-dithobenzoate functionality was aminolyzed to yield thiols that were simultaneously subjected to an exchange reaction with pyridyl disulfide at the chain ends, resulting in the formation of hyperbranched structures, which could proceed disulfide mediated degradation in the presence of reducing agent such as dl-Dithiothreitol (DTT), Tris(2-carboxyethyl)phosphine (TCEP) or glutathione. Biodegradable hyperbranched cationic polymers, poly(2-(dimethylamino)ethyl methacrylate) (PDMAEMA), have also been synthesized via RAFT mechanism for DNA binding and delivery [73].

**Figure 5:**
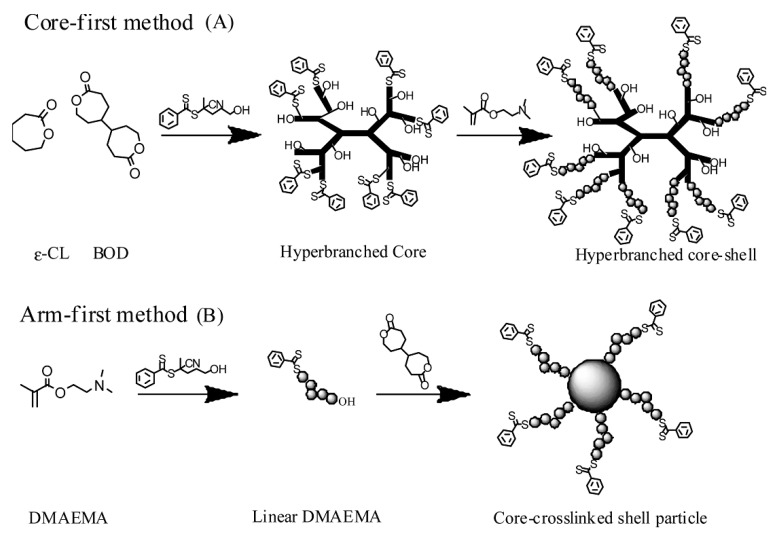
Scheme of degradable core–shell polymer via RAFT and ROP route. Routes (**A**,**B**) demonstrate the core-first method (for hyperbranched particles) and arm-first method (for CCS particles), respectively. Copyright 2011, American Chemical Society.

In addition to the biodegradable linkages, when the biodegradable polymers such as poly(lactide) (PLA) or polycaprolactone (PCL) are incorporated into cross-linked or self-assembled polymeric architectures, biodegradability can also be achieved. Schubert, Hoogenboom and coworkers synthesized a well-defined biodegradable macro-monomer, oligo(2-ethyl-2-oxazoline) methacrylate by direct end-capping of living oligo(2-ethyl-2-oxazoline) chains with in situ formed triethylammonium methacrylate, followed by homopolymerization via RAFT mechanism and then copolymerization using the homopolymer as macro-RAFT agent to achieve comb-like biodegradable architectures [74]. Despite the same combined polymerization techniques being used, different polymerization sequence may afford completely different polymeric structures. In research by Thurecht and coworkers, RAFT polymerization and ROP were used to synthesize both hyperbranched and microgel particles [75]. The core-first method afforded the hyperbranched core–shell structure, whereas the arm-first method gave core-cross-linked shell particles (Figure 5).

**Figure 6:**
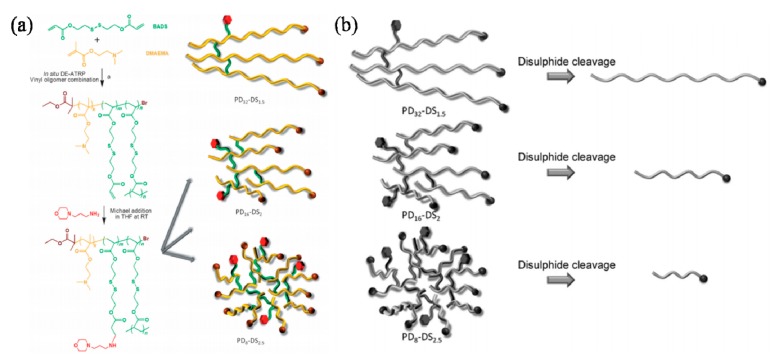
(**a**) (Left) Controlled radical cross-linking copolymerization through in situ DE-ATRP, followed by a post-functionalization process; and (Right) graphical representation of structures with different degrees of branching. The efficacy of functionalization depends on the content of the pendent vinyl groups; (**b**) Graphical representation of the degradation of structures with different degrees of branching. Copyright 2014, Wiley-VCH.

In addition to RAFT polymerization, ATRP was also used as a convenient tool for the synthesis of highly branched biocompatible poly(2-hydroxyethyl methacrylate), which was then used to prepare biocompatible fibers. The incorporation of partial disulfide-based dimethacrylate monomer in the polymerization conferred the biodegradability to the highly branched polymers [76]. Hedrick and coworkers described a new functional lactone containing a pendant acrylate group that can be of great interest for the design of new cross-linked biodegradable materials using combined ATRP and ROP techniques [77]. Similarly, Xu et al. reported the generation of comb-shaped copolymers composed of biocompatible hydroxypropyl cellulose backbones and cationic poly(2-dimethyl amino)ethyl methacrylate) side chains for gene delivery. The generated complex exhibited a stronger ability to bind with DNA, due to the increased surface cationic charges [78]. Comb-like and biodegradable supramolecular architectures can also be prepared using amphiphilic copolymer, poly(lactide)-*b*-poly(2-hydroxyethyl methacrylate) (PLA-*b*-PHEMA) with partially biodegradable PLA block and PHEMA biocompatible one using an orthogonal polymerization strategy via ROP and ATRP [79]. Likewise, the same methodology was also adopted to prepare ABA and star amphiphilic block copolymers composed of polymethacrylate bearing a galactose fragment and biodegradable poly(epsilon-caprolactone) [80]. Series of degradable branched poly(dimethylaminoethyl methacrylate) (PDMAEMA) copolymers were investigated by Zhao’s group. The branched PDMAEMA copolymers were synthesized by controlled radical cross-linking copolymerization. Efficient degradation processes were experimented for all of the copolymers. The degree of branching exhibited a big impact on the performance of transfection when tested on different cell types. The product with the highest degree of branching and highest degree of functionality had a superior transfection profile in terms of both transfection capability and the preservation of cell viability. The branched PDMAEMA copolymers show high potential for gene-delivery applications through a combination of the simplicity of their synthesis, their low toxicity and their high performance (Figure 6) [81].

### 2.3. Hydrogels and Nanogels

#### 2.3.1. Hydrogels

Hydrogels are optimal materials for tissue engineering scaffolds due to their tissue-like mechanical compliance and mass transfer properties. However, many hydrogels that have been widely used in medical science are not biodegradable, thus cannot be easily and quickly cleared out of the body. Therefore, using biocompatible and biodegradable co-polymers for fabricating hydrogels is much desired. Ratner and coworkers successfully prepared cross-linked nanogels of biodegradable poly(2-hydroxyethyl methacrylate) (PHEMA) as engineered tissue constructs using ATRP technique and an enzyme degradable cross-linking agent, polycaprolactone (PCL) and a degradable macro-initiator that also contained oligomeric PCL [37].

**Figure 7:**
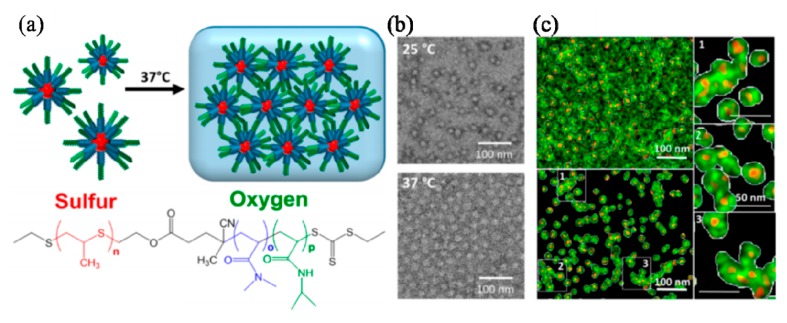
(**a**) Schematic representation of micelle gelation at 37 °C and polymer architecture coordinating with STEM-EDS element maps; (**b**) TEM images of PPS60-*b*-PDMA150-*b*-PNIPAAM150 micelles at 25 and 37 °C; (**c**) STEM-EDS element maps for sulfur (red) and oxygen (green) of PPS60-*b*-PDMA150-*b*-PNIPAAM150 core–shell compartments at 37 °C with image thresholding and background subtraction. Core-forming PPS produces the red signal for sulfur, while oxygen (appearing green) is present in the PDMA and PNIPAAM corona-forming blocks. Copyright 2014, American Chemical Society.

The hydrogel of nanostructured hyaluronic acid has also be generated in situ by Matyjaszewski’s group under physiological conditions (pH 7.4, 37 °C) by a combination of ATRP and Michael-type addition reaction using biodegradable nanogel precursors, 2-hydroxyethyl p(OEO300MA-*co*-methacrylate) (POEO300MA-*co*-PHEMA) [82]. RAFT agent in the form of “RAFT gel” has also been prepared by Takasu’s group via chemoselective polycondensations of a dicarboxylic acid containing a mercapto group and further used for the polymerization of methyl methacrylate to afford polyester containing biodegradable hydrogels [83]. It is well known that the synthetic poly(amino acid)s that have polypeptide backbone can be degraded in biological environments by enzymes such as proteinases and peptidases. Kubies et al. successfully prepared such cross-linked biodegradable hydrogels of a series of polymer architectures with the same polypeptide backbone via ring opening polymerization. They also found the enzyme-catalyzed hydrolysis can be controlled through copolymerization and/or side-chain modifications [38]. A combination of anionic and RAFT polymerization was used to synthesize an triblock polymer poly-[(propylenesulfide)-*b*-(*N*,*N*-dimethylacrylamide)-*b*-(*N*-isopropylacrylamide)] (PPS-*b*-PDMA-*b*-PNIPAAM) that forms physically cross-linked hydrogels when transitioned from mechanisms for reactive oxygen species (ROS) triggered degradation and drug release. At ambient temperature, PPS-*b*-PDMA-*b*-PNIPAAM assembled into 66 ± 32 nm micelles comprising a hydrophobic PPS core and PNIPAAM on the outer corona. The PPS-*b*-PDMA-*b*-PNIPAAM micelles were preloaded with the model drug Nile red and the resulting hydrogels demonstrated ROS-dependent drug release. The hydrogels were cyto-compatible in vitro and demonstrated to have utility for cell encapsulation and delivery. These hydrogels also possessed inherent cell-protective properties and reduced ROS-mediated cellular death in vitro. Subcutaneously injected PPS-*b*-PDMA-*b*-PNIPAAM polymer solutions formed stable hydrogels that sustained local release of the model drug Nile red for 14 days in vivo. These collective data demonstrate the potential use of PPS-*b*-PDMA-*b*-PNIPAAM as an injectable, cyto-protective hydrogel that overcomes conventional PNIPAAM hydrogel limitations such as syneresis, lack of degradability, lack of inherent drug loading and environmentally responsive release mechanisms (Figure 7) [84].

#### 2.3.2. Nanogels

Nanogels have drawn enormous attention due to their applications as targeted drug delivery scaffolds in biomedical science. Matyjaszewski’s group is pioneering the fabrication of hyperbranched polymeric architectures, particles, hydrogels and nanogels using ATRP strategies [85]. They reported the synthesis of stable biodegradable nanogels cross-linked with disulfide linkages using inverse miniemulsion ATRP methods. The biodegradation in the presence of glutathione tripeptide can trigger the release of encapsulated molecules including rhodamine 6 G, a fluorescent dye and doxorubicin (Dox), an anticancer drug, as well as facilitate the removal of empty vehicles [86]. They also prepared biodegradable nanogels as delivery carriers for carbohydrate drugs using ATRP in a cyclohexane inverse miniemulsion in the presence of a disulfide functionalized dimethacrylate cross-linker. These nanogels exhibited the high loading efficiency of rhodamine B isothiocyanate-dextran (RITC-Dx) exceeding 80% [87]. The same inverse miniemulsion ATRP strategy was also utilized to make biodegradable nanogels. Likewise, nanogels that can be degraded under various pH conditions were also prepared from biodegradable amphiphilic polymers synthesized by ATRP combined with ROP synthetic methodologies [88].

**Figure 8:**
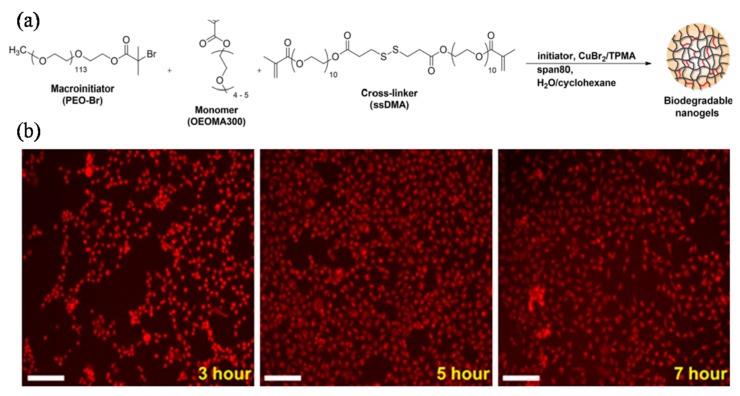
(**a**) Scheme for biodegradable nanogels synthesized by inverse miniemulsion ATRP; (**b**) Fluorescent images of cells with Ce6-loaded nanogels as a function of incubation time (scale bar 300 µm). Copyright 2016, Springer.

Recent advances in drug carrier design in the field of photodynamic therapy (PDT) have stimulated the development of numerous sophisticated drug delivery carriers. Kim and coworkers designed a novel biodegradable and biocompatible nanogels used as PDT carriers. The nanogels were synthesized through ATRP method using inverse miniemulsion and their biodegradability was determined in the presence of glutathione. The model photosensitizer (PS) was encapsulated in the biodegradable nanogels by simple mixing and sonication. The cellular uptake and the cytotoxicity of the nanogels before and after laser irradiation were determined. The results showed that the Ce6-loaded nanogels did not influence the cellular viability of the cells before light irradiation. Under light exposure, the Ce6-nanogel complex revealed strong photoactivity. These nanogels may enhance therapeutic efficacy of PSs without any complex chemical modifications with PSs (Figure 8) [89].

The stability of encapsulation in self-assembled system is usually limited by the requisite concentration for self-assembly formation. Once the encapsulation is achieved, the lack of targeting molecules on the drug carriers will compromise the efficiency for targeted delivery. To tackle this issue, Thayumanavan and coworkers successfully fabricated surface-functionalized polymer nanogels with facile hydrophobic guest encapsulation capabilities [90]. These biodegradable nanogels were first prepared from pyridyl disulfide pedant random copolymers that were prepared through RAFT mechanism via cross-linking through disulfide bonding, followed by the surface modification with a thiol-modified cell-penetrating peptide, Tat-SH. The internalization of Tat-SH modified nanogels occurred much more readily than that observed with the control gels, confirming the effectiveness of the modification of the nanogel surface. This presents a clear method for incorporating ligands onto the polymer nanoparticles and thus achieves specificity to pathogenic cells. Biodegradable nanogels/microgels have also been successfully prepared by RAFT polymerization using cross-linking agents that contain acid sensitive or disulfide intra-linkages. The surface tethered RAFT active centers allow further modifications and functionalizations via thiol-pyridyl disulfide exchange or thiol-ene reactions [91].

### 2.4. Micelles, Vesicles and Capsules

Polymers have been widely explored for the preparation of varied particles, e.g., micelles, vesicles and capsules, based on the expectation that these particles can be the appropriate reservoirs for controlled drug delivery. The advantage of using these polymer particles as drug carriers over traditional administration of free drugs lies in the increased circulation time in the body as these particles are usually big enough to prevent fast clearance through kidney filtration which has a cut-off molecular weight of 50,000 g·mol^−1^ [27]. Another advantage of polymer particles is called “stealth-like” effect which can be observed with the particles smaller than 200 nm or those surface decorated with specific polymers, e.g., poly(ethylene glycol) [92]. This “stealth-like” effect will greatly increase the circulation time. Polymer particles are usually prepared by amphiphilic block copolymers, where the hydrophobic block is used to form the core and the hydrophilic block from the corona in polar media. Research has revealed that the morphology of the polymeric particles might be mainly determined by the ratio of hydrophilic segment to the hydrophobic one [93]. Using block copolymers to prepare micelles has been extensively conducted and well-reviewed [8]. The design of polymeric particles with hydrophobic cores is based on the fact that anti-cancer drugs are usually hydrophobic and can be impregnated within the particle cores. Of course, the polymer particles can also be tailored with hydrophilic core and hydrophobic corona when required, mostly by manipulation of the polarity of preparation solvent. Once the drug is impregnated within particles, another issue arises with how to control the drug release. The traditional drug release from the particles is usually controlled by the self-degradation. However, if the polymers are designed as biodegradable, better control or more controlling means can be realized. The preparation of polymeric micelles for drug delivery using RAFT polymerization was reviewed by Stenzel [7]. In this section, we mainly discuss the preparation of polymer particles that can undergo biodegradation and their potential applications.

#### 2.4.1. Micelles

Micelles generated from well-defined diblock copolymers of thermoresponsive poly(*N*-isopropylacrylamide-*co*-*N*,*N*-dimethylacrylamide) blocks and biodegradable poly(d,l-lactide) blocks by the combination of RAFT polymerization and ROP were also prepared by Akimoto et al. The biodegradable polylactide (PLA) cores conferred the degradability to the micelles at acidic condition (pH 5.0). A much similar work was carried out by Zhu et al. who fabricated micelles using a thermal responsive poly(*N*-isopropylacrylamide) block, thus drug release could be thermally controlled. The presence of polycaprolactone (PCL) block makes the micelle biodegradable in biological environments [94].

**Figure 9:**
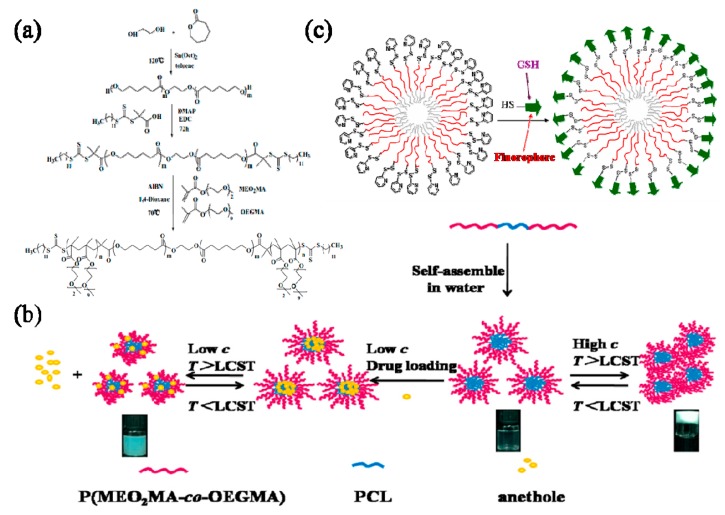
(**a**) Synthesis of triblock copolymer P(MEO_2_MA-*co*-OEGMA)-*b*-PCL-*b*-P(MEO_2_MA-*co*-OEGMA); (**b**) Schematic representation of the self-assembled thermo-sensitive core–shell micelles for temperature-stimulated drug release and gelation of tPBs. Copyright 2018, Elsevier; (**c**) Functionalized micelles and further attachment of fluorophores and targeting tripeptides.

In Ning’s study, well-defined, novel, linear, biodegradable and amphiphilic thermo-responsive ABA-type triblock copolymers, poly(2-(2-methoxyethoxy) ethyl methacrylate-*co*-oligo(ethylene glycol) methacrylate)-*b*-poly(*ε*-caprolactone)-*b*-poly(2-(2-methoxyethoxy) ethyl methacrylate-*co*-oligo (ethylene glycol) methacrylate) (P(MEO_2_MA-*co*-OEGMA)-*b*-PCL-*b*-P(MEO_2_MA-*co*-OEGMA)) (tBPs), were synthesized via a combination of ring-opening polymerization (ROP) of *ε*-caprolactone (*ε*CL) and RAFT polymerization of MEO_2_MA and OEGMA monomers. Thermo-responsive micelles were obtained through a self-assembly process of copolymers in aqueous medium. The hydrophobic drug of anethole was encapsulated in micelles through the dialysis method. The average particle sizes of drug-loaded micelles were determined by dynamic light scattering measurement. In vitro, the sustained release of the anethole was performed in pH 7.4 phosphate buffered saline at different temperatures. Results showed that the triblock copolymer micelles were quite effective in the encapsulation and controlled release of anethole. The vial inversion test demonstrated that the triblock copolymers could trigger the sol-gel transition which also depended on the temperature, and its sol-gel transition temperature gradually decreased with the concentration increasing (Figure 9a,b) [95].

Another issue arising with the particle delivered drug delivery is how to enhance the delivering efficiency. The unmodified particles are usually evenly distributed in the body, if this is the case side effect might happen. Therefore, achieving targeted drug delivery has attracted enormous interest. Davis and coworkers prepared surface functionalized micelles using amphiphilic triblock copolymers of oligo(ethyleneglycol) acrylate (PEG-A) and styrene (St), poly(PEG-A)-*b*-poly(St)-*b*-poly(PEG-A) by RAFT polymerization using a new bifunctional RAFT agent, *S*,*S*-bis[α, α′-dimethyl-α″-(2-pyridyl disulfide) ethyl acetate] trithiocarbonate (BDPET) [96]. These micelles were tailored with surface bound pyridyldisulfide (PDS) groups that are active to a free thiol group bearing model peptide, reduced glutathione, and a thiol modified fluorophore, rhodamine B, under mild reaction conditions (Figure 9c). It can be envisioned that, when these micelles are tailored with specific targeting molecules, the delivery efficiency should be greatly enhanced and the unwanted side effect can then be avoided. 

It is usually difficult to obtain the complicated polymer architectures using a single polymerization technique. Combining with organo-base catalyzed polymerization of l- or d-lactide Frey and coworkers, using ATRP technique, prepared biodegradable poly(isoglycerol methacrylate)-*b*-poly(l- or d-lactide) copolymer as building block for fabrication of spherical and large superamolecular vesicles via self-assembly in aqueous medium [97]. In most cases, the micelle cores are employed as drug reservoirs, however some novel micelles based on biodegradable poly (l-glutamic acid)-*b*-polylactide with paramagnetic Gd ions chelated to the shell layer were also prepared as a potential nanoscale magnetic resonance imaging (MRI)-visible delivery system [98].

In addition to RAFT polymerization, ATRP incorporating with ROP were also employed to synthesize a new class of supramolecular and biomimetic glycopolymer/poly(*ε*-caprolactone)-based polypseudorotaxane/glycopolymer triblock-copolymers. The polypseudorotaxane block was prepared by an inclusion reaction between biodegradable poly(*ε*-caprolactone) and α-cyclodextrin. These triblock biohybrids were then utilized to fabricate micelles or vesicles that possess hydrophilic glycopolymer shell and oligosaccharide threaded polypseudorotaxane core [99]. Likewise, quite similar biodegradable amphiphilic block copolymers with poly(γ-methyl-*ε*-caprolactone) (PmCL), o-nitrobenzyl (ONB) and polyacylic acid (PAA) blocks, and the same synthetic methodologies have also been prepared by Cabane et al. for fabrication of micelles as well. Furthermore, the as-fabricated micelles and vesicles are also photoresponsive due to the presence of a photodegradable OCN linker as a junction point between hydrophilic and hydrophobic chains [100].

Acetal is a pH sensitive group that is stable at pH 7 and prone to go hydrolysis at mild acidic pH of 4.0–5.0, with a half-life of 6.5 h, respectively. Zhong and coworkers [101] incorporated acetal groups into block copolymers comprising of a novel acid-labile polycarbonate and poly(ethylene glycol) (PEG) to generate pH-responsive biodegradable micelles as potential smart nano-vehicles for targeted delivery of anticancer drugs. Biodegradable cross-linked micelles were also prepared with a stimulus-responsive triblock copolymer synthesized via a bifunctional ATRP initiator containing intra-disulfide linkage [102]. When the micelles were prepared by stimulus-responsive copolymer and self-assembled on mica surface, pH manipulated switchable surface was achieved [103]. NMRP in combination with ROP were also utilized to prepare poly(*ε*-caprolactone-*b*-4-vinylpyridine) for preparation of micelles. As the so-prepared micelle impregnated a cationic core it can mediate the transportation of AuCl_4_^−^ anions from aqueous phase to the micelle core to afford micelle protected Au nanoparticles after reduction with NaBH_4_ [104].

#### 2.4.2. Vesicles

**Figure 10:**
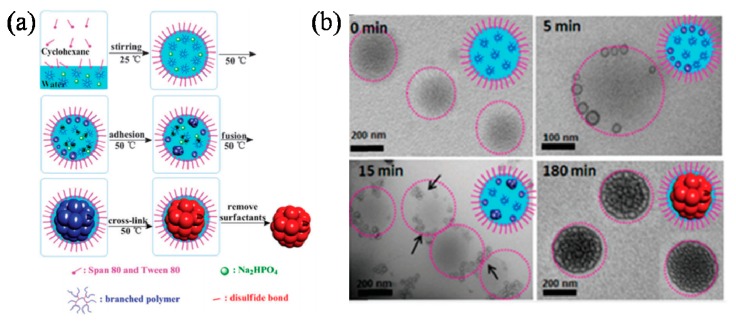
(**a**) Schematic outline of preparation of inverse emulsion nanodroplet templates containing the branched copolymer in the water phase and the possible mechanism for the formation of cross-linked large compound vesicles in nanodroplet templates; (**b**) TEM images of the self-assembled nanostructure of the branched copolymer in nanodroplet templates at different stages. Copyright 2014, Royal Society of Chemistry.

Polymeric vesicles or polymersomes are nano- or micrometer sized polymeric capsules with a bilayered membrane. Extensive applications can be envisioned in nanomedicine, in vivo diagnostics and drug delivery [6]. Du and Armes reported the facile preparation of vesicles in pure water medium using diblock copolymer, poly(*ε*-caprolactone)-*b*-poly[2-(methacryloyloxy) ethyl phosphorylcholine] (PCL-*b*-PMPC), which was synthesized using the combined methods of ROP, end-group modification and ATRP. These vesicles can be stabilized by sol-gel chemistry within the vesicle membrane [105]. In addition to the well-defined routine chemical polymerization methods, lipase-catalyzed condensation polymerization method was also used to synthesize biodegradable poly(10-hydroxydecanoic acid) (PHDA) and further modify it with ATRP initiator for grafting another hydrophobic polystyrene block for fabrication of polymeric nanoparticles in aqueous medium [106]. Vesicles can also be designed as pH sensitive for efficient DNA encapsulation and delivery, where the particles were prepared by poly(2-(methacryloyloxy)ethyl-phosphorylcholine)-*co*-poly(2-(diisopropylamino)ethyl methacrylate) (PMPC-*b*-PDPA) diblock copolymers. The PMPC block is highly biocompatible and nonfouling, while the PDPA block is pH-sensitive (pKa ~5.8–6.6, depending on the ionic strength) [107]. Wang and coworkers reported a novel method for the preparation of biodegradable large compound vesicles with controlled size and narrow size distribution by using aqueous nanodroplets as templates. PEG-based large compound vesicles (LCVs) were prepared through a self-assembly process of the temperature-responsive 2-(2-methoxyethoxy) ethyl methacrylate-oligo(ethylene glycol) methacrylate-*N*,*N*′-cystamine bisacrylamide (MEO_2_MA-OEGMA-CBA) branched copolymer. The sizes of the LCVs can be easily tuned by the amount of surfactants and the cross-linked reaction in LCVs occurred during the fusion process of small vesicles without any additional cross-linking agent. The formed LCVs are uniform, low toxic and resistant to nonspecific protein adsorption. The biodegradable and biocompatible LCVs can act as a vector for proteins (Figure 10) [108].

#### 2.4.3. Capsules

Multilayered polymer capsules assembled via layer-by-layer (LbL) technology have generated significant scientific and technological interest over the past decade because of their potential as advanced delivery and microreactor systems [109,110]. Caruso and coworkers are pioneering the preparation of versatile capsules via self-assembly for drug and gene delivery and controlled release, among which some of them are biodegradable [111,112,113,114]. For example, they fabricated low-fouling poly(*N*-vinyl pyrrolidone) (PVPON) capsules with engineered biodegradable properties via LbL process mediated by hydrogen bonding interaction. Due to the introduction of intra-disulfide linkages among the capsules they underwent destruction within 4 h in the presence of 5 mM glutathione. The cross-linked multilayers endowed the capsule with low-fouling properties to a range of proteins, including fibrinogen, lysozyme, immunoglobulin G, and bovine serum albumin [115]. Disulfide-stabilized poly(methacrylic acid) capsules that undergo reversible swelling in response to changes of external pH and degrade in the presence of a physiological concentration of glutathione were also prepared and investigated. 

**Figure 11:**
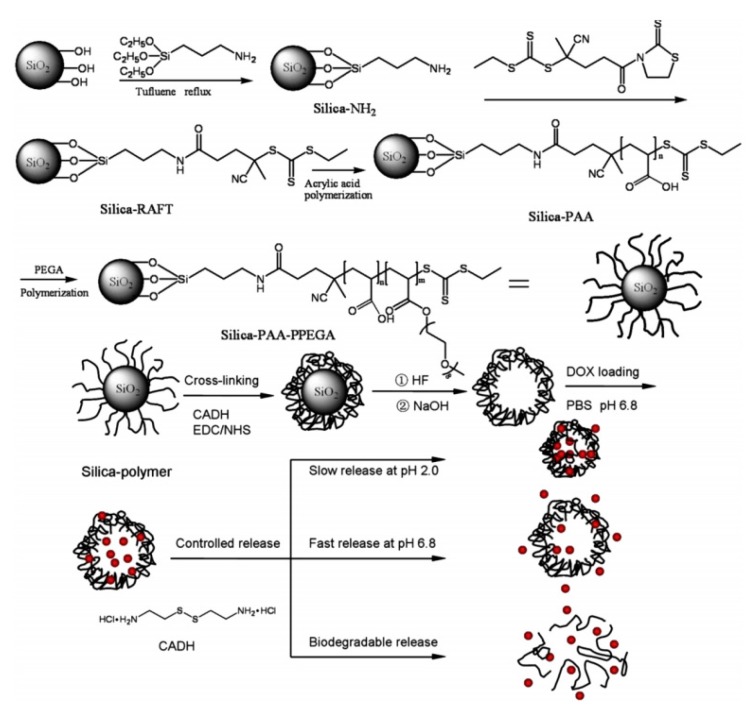
The preparation of pH responsive, biodegradable, biocompatible and cross-linked polymer capsules for controlled drug DOX (the red dots) release. Copyright 2014, Elsevier.

In Cui’s study, the preparation of pH responsive, biodegradable, biocompatible and cross-linked polymer capsules for controlled drug release was presented. The capsules were prepared using silica particles as templates for surface grafting of poly (acrylic acid) (PAA) and PAA-*co*-poly(polyethylene glycol) acrylate) (PAA-*co*-PPEGA) block copolymer via RAFT polymerization directly from silica particles, followed by cross-linking with cystamine dihydrochloride and removal of the silica template in the presence of hydrofluoric acid, respectively. The resultant polymer capsules were water soluble and biocompatible with a mean diameter of approximately 260 ± 10 nm. These polymer capsules were non-toxic to human cells at a low concentration, which are favorable to be utilized as drug carriers for pH responsive and biodegradation controlled drug release. Doxorubicin hydrochloride (DOX) was used as a model drug to test the drug loading and releasing properties of the polymer capsules. It was found that the DOX could be effectively loaded into the PAA and PAA-*co*-PPEGA capsules with a loading capacity up to 52.24% and 36.74%, respectively. The pH and biodegradation controlled release behaviors of DOX loaded PAA-PPEGA capsules were also explored. The results implied that both PAA and PAA-*co*-PPEGA capsules are promising platforms for pH and biodegradation controlled drug delivery systems, while the PAA-*co*-PPEGA capsules exhibit less cytotoxicity (Figure 11) [116].

### 2.5. Polymeric Architectures Based on Biodegradable Synthetic or Natural Precursors

**Figure 12:**
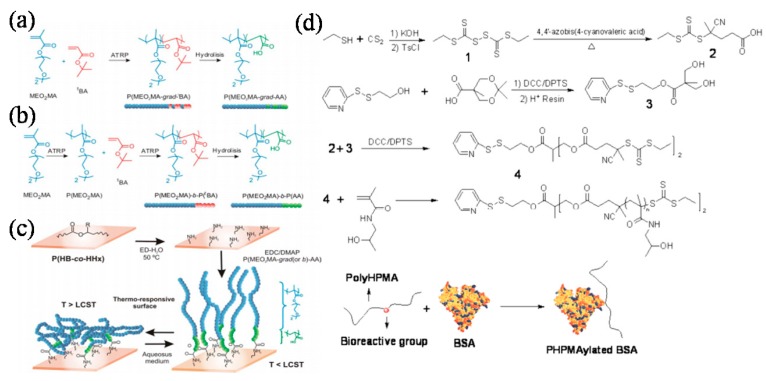
(**a**) Synthesis of the gradient copolymers; (**b**) Synthesis of the block copolymers; (**c**) Synthesis of thermo-responsive surfaces. Copyright 2010, Wiley-VCH; (**d**) Synthesis of branched poly (*N*-(2-hydroxypropyl) methacrylamide) (PHPMA) and the subsequent conjugation with protein. Copyright 2009, American Chemical Society.

Biodegradable architectures can also be achieved by grafting polymer chains onto the biodegradable precursors. The degradation of biodegradable precursors will consequently disintegrate the as-prepared architectures. These precursors can be synthetic biocompatible and biodegradable films, such as poly(3-hydroxybutyrate-*co*-3-hydroxyhexanoate) (P(HB-*co*-HHx)) (Figure 12a) [117]. Cellulose is a natural polysaccharide consisting of a linear chain of several hundreds to over ten thousand linked d-glucose units. It is the major constituent of paper, paperboard, and card stock and of textiles made from cotton, linen, and other plant fibers. Its high hydrophilicity is right due to the multi-hydroxy groups from the glucose units. These hydroxy groups not only make the cellulose chains holding firmly together side-by-side and forming microfibrils with high tensile strength, they can also be used to make soluble and functionalized cellulose. The biodegradable cellulose would be a good precursor for generation of biodegradable architectures. A few groups have explored the possibility of modifying cellulose. Carlmark et al. successfully modified the cellulose using ATRP via “graft from” methodology. They first attached 2-bromoisobutyryl bromide on the cellulose surface through the condensation reaction with the surface hydroxyl group, followed by the ATRP reaction to create an amphiphilic block copolymer layer on it [118].

Using the same methodology, Perrier and coworkers successfully attached different RAFT agents through the surface hydroxyl groups for direct grafting an amphiphilic copolymer, poly(ethylene glycol)-*b*-poly(*l*-lactic acid), from the cellulose surface. The biodegradable poly(l-lactic acid) block further facilitates the biodegradability of the so-prepared architecture [119]. One advantage of RAFT polymerization is the versatile initiation methods. In addition to the commonly used thermal initiation, other ionizing radiation sources, such as γ-ray, ultraviolet, microwave and X-ray radiation, have also been used to initiate RAFT controlled polymerizations [20,120,121,122]. Barsbay and coworkers used both thermal and γ-ray initiations and RAFT polymerization to modify cellulose with styrene and sodium 4-styrenesulfonate polymeric brushes using “graft from” methodology [123,124]. Cellulose fiber was also modified with biodegradable polyesters by the aid of host-guest inclusion complexation between β-cyclodextrin and adamantine motifs [125].

“Glycopolymers”, particularly the multivalent ones have attracted tremendous attention due to the potential applications in biomedicine and biomaterials. Dong and coworkers synthesized a four-armed star glycopolymer composed of block copolymer arms bearing lactone end groups. These star polymers could self-assemble onto nanoparticles that carry the lactose groups on their surface, allowing for the further complexing with lectins to achieve biodegradable biohybrids [126]. Glycopolymers were synthesized by Stenzel and coworkers using RAFT polymerization and thio-ene click chemistry to fabricate glucose surface tethered glycomicelles for further complexation with concanavalin A, a mannose and glucose specific lectin. These biodegradable and biocompatible glycomicelles could be utilized as potential drug carriers [127]. Qiu et al. also prepared large spherical micelles in aqueous solution, using star-shaped polypeptide/glycopolymer biohybrids composed of poly(γ-benzyl l-glutamate) and poly(d-gluconamidoethyl methacrylate) prepared via ROP and ATRP. The generated micelles had a helical polypeptide core surrounded by a multivalent glycopolymer shell, which potentially provides a platform for fabricating targeted anticancer drug delivery system and for studying the glycoprotein functions in vitro [128].

In contrast with the polymer–drug conjugates prepared thus far, in which the drug is typically attached via an enzymatically or hydrolytically cleavable linker, Apostolovic’s group reported the noncovalent polymer therapeutics based on a conceptually novel class of polymers prepared using RAFT mechanism. The polymer backbone was used to attach the cargo via a noncovalent, biologically inspired coiled coil linker, which was formed by heterodimerization of two complementary peptide sequences that are linked to the polymer carrier and the cargo, respectively [129].

### 2.6. Biodegradable Biomolecule-Polymer Conjugates

Bioconjugates refer to a category of polymer conjugates with widespread biomolecules, which have attracted increasing interest as they have numerous potential applications in biotherapeutics, bioseparation and functional materials field. The importance of bioconjugates lies in the fact that the biomolecules will exhibit prolonged circulation time in biofluids [130,131] and their immunogenicity and antigenicity can also be reduced by the incorporation of biocompatible polymer fragments [2,132,133]. When the bioconjugates are designed with biodegradable linker between the biomolecules and the polymer fragments these biomolecules can be released in vivo, therefore, their bioactivities can be reversed [20,134]. On the other hand, most biomolecules, e.g., proteins and enzymes, consist of peptides that are linked by biodegradable disulfide bonding. In this case, these biomolecules are also biodegradable, making the whole bioconjugates biodegradable. 

Davis and coworkers delivered elegant research on the preparation of biodegradable conjugates. Free thiol tethered biomolecule, e.g., bovine serum albumin (BSA), has been successfully modified with several polymers to afford biodegradable homo- or hetero-bioconjugates under ambient condition using room temperature initiation via RAFT polymerization (Figure 12d) [20,135,136,137,138]. By tailoring the bioconjugates with disulfide linkage between lysozyme and the polymer chains, the bioactivity of lysozyme can be reversed during the biodegradation process [134]. They have also successfully modified lysozyme with well-defined poly-*N*-(2-hydroxypropyl) methacrylamide via surface modifications through amide bonding to tailor the enzyme’s bioactivity [139]. A latest study reported the modification of fragile glucose oxidase (GOx) with biocompatible polymer, poly(ethyleneglycol) acrylate (polyPEG-A) and thermoresponsive copolymer of poly(ethyleneglycol) acrylate and di(ethyleneglycol) ethyle ether acrylate [poly(PEG-A-*co*-DEG-A)] to afford biodegradable enzyme–polymer conjugates. Bio-cleavage of the polymer chains from the GOx surface obviously recovered the enzymatic activity [62]. These smart enzyme–polymer conjugates would envision promising applications in biotechnology and biomedicine. Maynard and coworkers also achieved significant advances in the preparation of bioconjugates using either ATRP or RAFT polymerization. They successfully modified si-RNA with a biodegradable polymer fragment. Since *si*-RNA is considered an effective targeting molecule, its biodegradable polymer conjugates could be good candidates for potential bio-therapeutics [140]. In addition to RAFT polymerization, ATRP has also been successfully applied to prepare biodegradable polymer conjugates with BSA [141,142] and engineered lysozyme [143].

## 3. Conclusions and Perspectives

This review has discussed the synthesis and applications of biodegradable polymeric architectures using different RDRPs. These biodegradable polymeric structures can be designed as well-defined star-shaped, cross-linked or hyperbranched, through which more complicated nanoparticles such as micelles, vesicles and capsules can be fabricated via either self-assembly or cross-linking methodologies. Nanogels and hydrogels can also be prepared via RDRPs. Their applications in biomedical science are also discussed. Biodegradable polymeric architectures can be prepared with both synthetic and natural precursors.

As discussed in this review, RDRPs have proven to be convenient tools for the synthesis of the versatile biodegradable polymeric architectures to meet varied applications. Driven by the practical application and commercialization, the design of more complicated polymeric architectures with controllable biodegradability will be expected. However, it is worth noting that a fast biodegradable process in vivo is not desired in some situations. Therefore, designing and fabricating the polymeric architectures with controllable and slow biodegradability would be a critical issue in this field. To achieve this, many other different polymerization techniques are required besides RDRPs.
